# The Global Burden of Sepsis and Septic Shock

**DOI:** 10.3390/epidemiologia5030032

**Published:** 2024-07-25

**Authors:** Luigi La Via, Giuseppe Sangiorgio, Stefania Stefani, Andrea Marino, Giuseppe Nunnari, Salvatore Cocuzza, Ignazio La Mantia, Bruno Cacopardo, Stefano Stracquadanio, Serena Spampinato, Salvatore Lavalle, Antonino Maniaci

**Affiliations:** 1 Department of Anaesthesia and Intensive Care, University Hospital Policlinico “G. Rodolico-San Marco”, 24046 Catania, Italy; 2 Department of Biomedical and Biotechnological Sciences, University of Catania, Via Santa Sofia 97, 95123 Catania, Italy; giuseppe.sangiorgio@phd.unict.it (G.S.); stefania.stefani@unict.it (S.S.); s.stracquadanio@unict.it (S.S.); 3 Unit of Infectious Diseases, Department of Clinical and Experimental Medicine, ARNAS Garibaldi Hospital, University of Catania, 95123 Catania, Italy; andrea.marino@unict.it (A.M.); giuseppe.nunnari1@unict.it (G.N.); cacopard@unict.it (B.C.); serenaspampinato93@gmail.com (S.S.); 4 Department of Medical, Surgical Sciences and Advanced Technologies “GF Ingrassia” ENT Section, University of Catania, 95123 Catania, Italy; s.cocuzza@unict.it (S.C.); igolama@gmail.com (I.L.M.); 5 Department of Medicine and Surgery, University of Enna “Kore”, 94100 Enna, Italy; salvatore.lavalle@unikore.it (S.L.); antonino.maniaci@unikore.it (A.M.)

**Keywords:** sepsis, septic shock, global burden, epidemiology, risk factors

## Abstract

A dysregulated host response to infection causes organ dysfunction in sepsis and septic shock, two potentially fatal diseases. They continue to be major worldwide health burdens with high rates of morbidity and mortality despite advancements in medical care. The goal of this thorough review was to present a thorough summary of the current body of knowledge about the prevalence of sepsis and septic shock worldwide. Using widely used computerized databases, a comprehensive search of the literature was carried out, and relevant studies were chosen in accordance with predetermined inclusion and exclusion criteria. A narrative technique was used to synthesize the data that were retrieved. The review’s conclusions show how widely different locations and nations differ in terms of sepsis and septic shock’s incidence, prevalence, and fatality rates. Compared to high-income countries (HICs), low- and middle-income countries (LMICs) are disproportionately burdened more heavily. We talk about risk factors, comorbidities, and difficulties in clinical management and diagnosis in a range of healthcare settings. The review highlights the need for more research, enhanced awareness, and context-specific interventions in order to successfully address the global burden of sepsis and septic shock.

## 1. Introduction

A dysregulated host response to infection resulting in organ dysfunction is the hallmark of sepsis, a potentially fatal illness [1]. The intricate syndrome arises from the interaction between the virus that is infecting the host and the immune system [2]. Updated definitions for sepsis and septic shock were released in 2016 with the release of the Third International Consensus Definitions for Sepsis and Septic Shock (Sepsis-3) [3]. Sepsis is described by Sepsis-3 as a dysregulated host response to infection resulting in life-threatening organ malfunction; septic shock is a subtype of sepsis characterized by circulatory and cellular/metabolic dysfunction that is associated with an increased risk of death [3].

Each year, sepsis and septic shock claim millions of lives globally and constitute a substantial global health burden [4]. Sepsis continues to be a major cause of morbidity and death worldwide, despite improvements in medical care and raised awareness [5]. Sepsis has been declared a worldwide health concern by the World Health Organization (WHO), which has also advocated for coordinated measures to lessen its prevalence [6]. Sepsis is not as common in all demographics and geographical areas; rates are higher in low- and middle-income countries (LMICs) than in high-income countries (HICs) [7]. The greater incidence of sepsis in LMICs is caused by a number of factors, including limited access to healthcare, delayed diagnosis and treatment, and the predominance of infectious illnesses [8]. The aging population, rising rates of invasive operations, and the emergence of bacteria resistant to antibiotics have all contributed to the ongoing prevalence of sepsis in HICs [9]. Sepsis has long-term effects that go beyond the acute stage of the disease. Sepsis survivors frequently suffer from chronic physical, mental, and cognitive deficits that lower their quality of life and increase their need for medical treatment [10]. Sepsis has a significant financial impact due to the high expenses of acute care, long-term rehabilitation, and lost productivity [11]. In order to influence public health policies and clinical practice, it is imperative to comprehend the epidemiology, risk factors, pathophysiology, and therapeutic techniques of sepsis and septic shock, given their substantial global burden [7,12,13]. In addition to highlighting the prospects and obstacles for improving patient outcomes and lowering the overall burden of this debilitating condition, this thorough review attempts to give an in-depth overview of the present state of knowledge regarding the global burden of sepsis and septic shock.

## 2. Methods

To find pertinent research on the worldwide burden of sepsis and septic shock, a thorough literature search was carried out. Major electronic databases such as PubMed, Embase, Scopus, and Web of Science were used in the search. The Medical Subject Headings (MeSH) phrases for sepsis, septic shock, epidemiology, incidence, prevalence, mortality, and global burden were combined with keywords in the search method. Furthermore, manual searches of pertinent review articles and reference lists from included research were carried out to find any other studies that might have been overlooked during the electronic database search. The following criteria were met by the included studies: (1) original research articles, systematic reviews, or meta-analyses; (2) studies that addressed the epidemiology, incidence, prevalence, or mortality of sepsis or septic shock; (3) data on the burden of sepsis or septic shock at the national, international, or regional levels; and (4) English-language publications. Case series, editorials, opinions, conference papers, or case reports were not included. To keep the focus on the general global burden of sepsis and septic shock, studies that only looked at particular subpopulations (like neonatal sepsis and maternal sepsis) or specific infections (like COVID-19-related sepsis) were also disregarded. Based on the inclusion and exclusion criteria, the titles and abstracts of the identified studies were evaluated by two independent reviewers. Potentially eligible studies’ full texts were located and examined before being finally included. When there were disagreements among the reviewers, they were settled by consensus-building and, if required, by consulting a third reviewer. The information on study characteristics (e.g., authors, year of publication, study design, and sample size), study population, sepsis and septic shock definitions used, incidence and prevalence rates, mortality rates, and any other pertinent findings were extracted from the included studies using a standardized data extraction form. The AMSTAR 2 tool for systematic reviews and meta-analyses was among the acceptable instruments used to evaluate the quality of the included studies, taking into account the study type [14]. In light of the expected variety in study designs, populations, and outcomes, the retrieved data were synthesized using a narrative approach. The primary goal of the synthesis was to provide a broad picture of the incidence, prevalence, and mortality rates of sepsis and septic shock worldwide across various nations and regions. When possible, subgroup analyses based on variables including age, gender, and healthcare environment were carried out. Along with highlighting any discrepancies, trends, or patterns in the prevalence of sepsis and septic shock, the synthesis also pointed up any knowledge gaps that need to be filled with additional study. 

## 3. How Did the Definition of Sepsis Change over Time?

The definition of sepsis has evolved over time as our understanding of the complex pathophysiology and clinical manifestations of this condition has advanced. In 1991, the first consensual definition of sepsis was introduced, which defined sepsis as a systemic inflammatory response syndrome (SIRS) caused by infection [15]. This definition, however, was criticized for being overly sensitive and lacking specificity, as SIRS criteria could be met in many non-infectious conditions [16]. In 2001, a second consensual definition (Sepsis-2) was proposed, which maintained the SIRS criteria but emphasized the presence of organ dysfunction as a key feature of severe sepsis [17]. The Sepsis-2 definition also introduced the concept of septic shock as a subset of sepsis with circulatory and cellular/metabolic abnormalities profound enough to substantially increase mortality [17]. In 2016, the Third International Consensus Definitions for Sepsis and Septic Shock (Sepsis-3) were released, which represented a significant shift in the conceptualization of sepsis [1]. The Sepsis-3 definition abandoned the SIRS criteria and defined sepsis as a life-threatening organ dysfunction caused by a dysregulated host response to infection [1]. This definition emphasized the importance of organ dysfunction and the role of the host response in the pathogenesis of sepsis. Septic shock was redefined as a subset of sepsis with circulatory and cellular/metabolic dysfunction associated with a higher risk of mortality [1]. The Sepsis-3 definition also introduced the quick Sequential Organ Failure Assessment (qSOFA) score as a bedside tool to rapidly identify patients with suspected infection who are at risk of poor outcomes [1]. The evolution of the sepsis definition reflects the growing understanding of the complex pathophysiology of this condition and the need for more precise and clinically relevant diagnostic criteria.

## 4. What Is the Epidemiology of Sepsis and Septic Shock?

The incidence rates of sepsis and septic shock vary throughout continents and countries, making them serious global health concerns [5]. According to a recent analysis by the Global Burden of Disease Study 2017, there were 11.0 million sepsis-related fatalities and 48.9 million instances of sepsis globally in 2017 [5]. More recently, a meta-analysis by Fleischmann-Struzek et al. reported an estimated mortality rate of 26.7% among this population of patients, with a 46% increase of incidence after 2008 [18]. Sepsis has an age-standardized incidence rate of 677.5 cases per 100,000 people worldwide, with sub-Saharan Africa, Oceania, and South Asia having the highest rates [5]. Sepsis and septic shock incidence rates differ markedly by continent. Between 500 and 1000 cases of sepsis occur in North America for every 100,000 people, with the United States having greater rates than Canada [19,20]. The incidence rate in Europe is estimated to be between 400 and 800 cases per 100,000 people [21,22]. On the other hand, sepsis is more common in low- and middle-income countries (LMICs), with rates in some Asian and African nations surpassing 1500 cases per 100,000 people [23,24,25]. Sepsis- and septic-shock-related mortality rates also range significantly between geographical areas. Sepsis mortality varies from 15% to 25% in high-income countries (HICs), whereas septic shock mortality might reach 30% to 40% [26,27]. Nonetheless, mortality rates are significantly greater in LMICs, frequently surpassing 40% in sepsis and 50% in septic shock [28,29]. Sepsis death rates have improved over time, especially in HICs, according to trends. Sepsis death rates in the US have decreased recently, from over 35% in the early 2000s to 15–20% [30,31]. Comparable patterns have been seen in European nations, where declining death rates have been credited to advancements in supportive care, timely administration of the right antibiotics, and enhanced early detection [31,32]. Sepsis mortality rates in low- and middle-income countries (LMICs) have remained high throughout time, indicating the need for better access to healthcare resources and the application of evidence-based management practices [8,33]. There are notable differences in the prevalence of sepsis and septic shock between industrialized and poor nations. Sepsis is less common in HICs, but the total burden is still significant because of the aging population and rising rates of chronic comorbidities [12,34]. Sepsis in these contexts is more frequently linked to invasive procedures and illnesses related to healthcare [35]. On the other hand, sepsis and septic shock are disproportionately more common in LMICs [7,36]. Higher prevalence of infectious diseases, restricted access to healthcare services, poor sanitation and hygiene, and delayed presentation to medical facilities are some of the factors causing this increased burden [37,38]. Sepsis in these conditions is more often brought on by community-acquired infections such malaria, pneumonia, and diarrheal illnesses [39,40]. The differences in the prevalence of sepsis between developed and underdeveloped nations highlight the necessity of focused therapies and resource distribution. HICs prioritize improving sepsis care and lowering infections linked to healthcare, but LMICs need a more all-encompassing strategy that tackles the underlying social determinants of health, fortifies healthcare systems, and enhances access to vital medical resources [41,42]. Severe sepsis and septic shock continue to be major global health system concerns. For the purpose of directing public health policy and clinical practice, it is essential to comprehend the worldwide epidemiology of sepsis, including incidence rates, mortality trends, and differences between industrialized and developing nations. In places with limited resources, efforts to lower the worldwide burden of sepsis should concentrate on early detection, the timely start of evidence-based treatments, and addressing the underlying social determinants of health [8]. The necessity for context-specific therapies and research is highlighted by the differences in sepsis epidemiology between geographical areas. Priorities in HICs include lowering healthcare-associated infections, improving sepsis management, and tackling the problems brought on by an aging population and rising comorbidities [9,11]. The implementation of cost-effective sepsis management strategies, bolstering infection prevention and control measures, and enhancing access to healthcare services should be the main priorities in LMICs [36]. Moreover, there is a need for the general public and healthcare professionals to be better informed about sepsis [17]. Appropriate care should be started as soon as possible after sepsis signs and symptoms are recognized, since this can greatly enhance patient outcomes [31,39]. The goals of educational programs and public health campaigns should be to raise awareness of sepsis and encourage prompt care-seeking behavior. The complicated biology of sepsis should be further understood, new prognostic and diagnostic biomarkers should be found, and creative treatment approaches should be developed. Particularly in environments with limited resources, collaborative international research projects can aid in filling in knowledge gaps and making it easier to apply research findings to clinical practice (Table 1) [33]. 

## 5. What Are the Risk Factors and Comorbidities across Continents?

**Figure 1:**
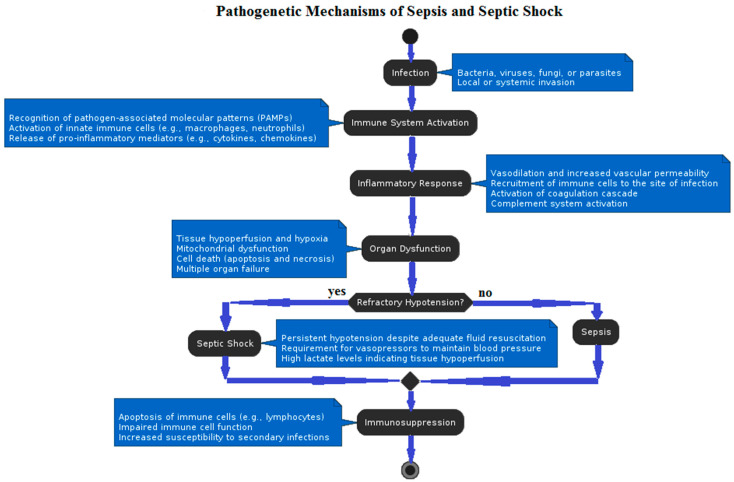
Flow diagram describing pathogenetic mechanisms underlying sepsis and septic shock.

Demographic factors, such as age and sex, play a significant role in the global burden of sepsis. Advanced age is a well-established risk factor for sepsis, with the incidence and mortality rates increasing substantially in older populations [12,43]. The aging population in many countries, particularly in high-income countries, contributes to the growing burden of sepsis [44]. Sex differences in sepsis incidence and outcomes have also been reported, with some studies suggesting a higher incidence in males but a higher risk of mortality in females [45,46] (Figure 1).

Chronic health conditions, such as diabetes, cardiovascular disease, chronic obstructive pulmonary disease (COPD), and cancer, are major risk factors for sepsis [47,48]. The increasing prevalence of these conditions worldwide, driven by factors such as aging populations, obesity, and lifestyle changes, contributes to the growing burden of sepsis [49]. Patients with chronic health conditions are more susceptible to infections and have a higher risk of developing sepsis and experiencing adverse outcomes [50,51]. Acute medical and surgical conditions are significant contributors to the global burden of sepsis. Pneumonia, urinary tract infections, and intra-abdominal infections are among the most common sources of sepsis [52,53]. However, the site of infection can significantly influence the outcomes of sepsis. Respiratory infections, particularly pneumonia, are the most common source of sepsis and are associated with higher mortality rates compared to other sites of infection [50]. Abdominal infections, such as peritonitis and intra-abdominal abscesses, are also associated with increased mortality and often require surgical intervention in addition to antimicrobial therapy [39]. Urinary tract infections, while common, generally have lower mortality rates compared to respiratory and abdominal infections [53]. Soft tissue infections, including cellulitis and necrotizing fasciitis, can lead to severe sepsis and septic shock, with necrotizing fasciitis having particularly high mortality rates [53]. Bloodstream infections, especially those caused by multidrug-resistant organisms, are associated with high morbidity and mortality, and their outcomes are heavily influenced by the timing and appropriateness of antimicrobial therapy [51,52]. Surgical procedures, particularly in emergency settings or in patients with underlying comorbidities, also increase the risk of sepsis [54]. Trauma, burns, and other acute injuries can lead to sepsis, especially in resource-limited settings where timely and appropriate care may be lacking [55,56]. Environmental and socioeconomic factors play a crucial role in the global burden of sepsis. Poverty, inadequate sanitation, and limited access to clean water and healthcare services contribute to the higher incidence of sepsis in low- and middle-income countries [57,58]. Overcrowding, malnutrition, and exposure to indoor air pollution further increase the risk of infections and sepsis in these settings [59]. In high-income countries, socioeconomic disparities, such as low income and education levels, are associated with a higher risk of sepsis and worse outcomes [60,61]. Genetic factors may influence the susceptibility to sepsis and its outcomes across different populations. Variations in genes involved in the immune response, such as those encoding cytokines, toll-like receptors, and coagulation factors, have been associated with the risk of sepsis and its severity [62,63]. However, the role of genetic factors in sepsis susceptibility and outcomes remains complex and not fully understood, with inconsistent findings across studies and populations [64]. Further research is needed to elucidate the interplay between genetic factors, environmental influences, and other risk factors in determining sepsis susceptibility and outcomes across different populations.

## 6. What Are the Differences in Diagnosis and Clinical Management across Various Healthcare Settings?

Improved patient outcomes and prompt commencement of appropriate treatment depend on the early detection and diagnosis of sepsis. Nonetheless, there are many obstacles in this area for environments with low resources [8]. Patients with sepsis may not seek medical attention until later if they have limited access to healthcare services, especially in rural areas [58]. Early detection of sepsis is made more difficult by the lack of qualified healthcare professionals, such as doctors and nurses [65]. It is possible that medical professionals working in these environments lack sufficient training to recognize sepsis symptoms and signs, which could result in missed or delayed diagnosis [32]. In settings with low resources, effective diagnosis of sepsis is further hampered by inadequate diagnostic instruments and laboratory services [66]. It is difficult to determine the etiological pathogen and direct focused antibiotic therapy due to a paucity of trustworthy microbiological testing, such as blood cultures and other diagnostic procedures [67]. Point-of-care diagnostic tests have limited utility in these situations because they are frequently unavailable or prohibitively expensive, such as lactate monitoring and biomarker assays [68]. In resource-constrained settings, clinical scoring systems, like the rapid Sequential Organ Failure Assessment (qSOFA), have been proposed as straightforward bedside tools to identify patients at risk of sepsis and poor outcomes [1]. The usefulness of qSOFA in these circumstances is still up for dispute, while some research indicates that it might not function as effectively as it would in high-income nations [69,70]. Clinical scoring systems are not widely adopted because they have not been validated in many healthcare situations and demographics [71]. The creation of context-specific clinical guidelines, the adoption of easy-to-use, low-cost diagnostic instruments, and healthcare professional education and training should be the main focuses of strategies to enhance early detection and diagnosis of sepsis in settings with limited resources [72]. Healthcare professionals’ capacity to identify and treat sepsis can be improved by incorporating sepsis education into their continuing education and medical and nursing curriculum [73]. The early detection of sepsis and the direction of management decisions can be aided by the creation and validation of clinical decision support tools tailored to the regional environment [74]. The foundations of sepsis care include source control and prompt administration of suitable antibiotic medication [75]. Nonetheless, there are significant differences in these therapies’ accessibility and availability between various healthcare settings. Access to vital antimicrobials, especially broad-spectrum antibiotics, may be restricted in environments with limited resources for a number of reasons [76]. In areas with low resources, the cost of antimicrobials can provide a substantial obstacle to their accessibility. It is possible that many patients in these situations cannot afford the antibiotics they need, which could cause treatment to be delayed or insufficient [76]. This issue is made worse by the lack of health insurance and the insufficient public support for healthcare [77]. In resource-constrained environments, supply chain problems, such as insufficient medicine distribution, storage, and procurement processes, might result in shortages of vital antimicrobials [78]. Antibiotic quality and effectiveness can be impacted by unreliable cold chain storage and delivery, especially in rural and isolated settings [79]. Effective antimicrobial therapy may not always be readily available in places with limited resources due to regulatory obstacles such as the unregistered status of some antibiotics or the prevalence of counterfeit medications [80]. Because resistant organisms may be more common in these situations, the growing threat of antibiotic resistance makes the choice of an appropriate empirical therapy even more difficult [81,82]. In order to effectively manage sepsis brought on by certain diseases, such as intra-abdominal or soft tissue infections, source control measures such as surgical debridement or abscess drainage are essential [83]. However, access to timely and suitable source control may be restricted in environments with limited resources [83,84]. These interventions may be delayed or impossible to carry out in the absence of surgical facilities, qualified staff, and required equipment [85]. In resource-constrained contexts, strategies to enhance the availability and accessibility of antimicrobial therapy and source control should prioritize bolstering drug procurement and distribution networks, guaranteeing sufficient funding for critical medications, and enhancing surgical care accessibility [86]. Optimizing the use of existing antibiotics and preventing the formation of resistance can be achieved through the implementation of antimicrobial stewardship programs tailored to the local environment [87]. Other crucial actions include teaching medical professionals how to identify and handle source control problems and incorporating source control into sepsis management protocols [88]. Although fluid resuscitation and hemodynamic support are essential parts of sepsis therapy, regional variations exist in these techniques [41]. Early goal-directed treatment (EGDT) was formerly extensively advocated in high-income nations as a method to direct resuscitation in sepsis [89]. In order to accomplish particular hemodynamic goals, EGDT included focused interventions, central venous catheterization, and continuous monitoring of central venous oxygen saturation [90]. Subsequent large-scale trials, including the ProCESS, ARISE, and ProMISe trials, conducted in high-income settings, failed to show that EGDT was superior to standard treatment [91,92,93]. These results prompted a move away from protocolized EGDT and toward a more customized resuscitation strategy [94]. The concepts of early detection, fluid resuscitation, and vasopressor support are still crucial to the treatment of sepsis in high-income nations, even in the absence of evidence for EGDT [39]. According to the guidelines of the Surviving Sepsis Campaign, the first three hours of fluid resuscitation should be focused on providing 30 mL/kg of crystalloids [95]. Patients who do not respond to appropriate fluid resuscitation are advised to use vasopressors, such as norepinephrine [96]. The recommendations also stress the significance of routinely reevaluating hemodynamic status and using dynamic measurements to direct fluid therapy, such as passive leg lifts or variations in pulse pressure [97]. Resuscitation techniques may need to be modified in environments with low resources because of the possible lack of access to IV fluids, monitoring equipment, and vasopressors [8]. The inability to direct resuscitation based on hemodynamic parameters may be hampered by the absence of sophisticated monitoring tools, such as arterial lines or central venous catheters [98]. Alternative fluids, such as colloids or hypotonic solutions, may be used as a result of the restricted supply of intravenous fluids, especially balanced crystalloids [99]. In environments with limited access to intensive care assistance and mechanical breathing, questions have been raised regarding the possible harm that vigorous fluid resuscitation may cause [100]. Children with acute fever illness and poor perfusion were shown to have a higher death rate when receiving fluid bolus therapy, according to the results of the FEAST experiment, which took place in sub-Saharan Africa [101]. These results emphasize the necessity of exercising prudence and evaluating fluid responsiveness with great care in environments with restricted resources [42]. In resource-constrained settings, strategies to maximize hemodynamic support and fluid resuscitation should center on using easily accessible, low-tech monitoring methods, including inferior vena cava ultrasonography or capillary refill time, to guide therapy [102]. It is also critical to design context-specific resuscitation guidelines that consider the hazards associated with over-resuscitation as well as the resources at hand [100]. To enhance results, healthcare professionals must be trained in the proper administration of fluids and vasopressors, as well as the identification and treatment of fluid overload [30]. Although their application varies depending on the context, adjunctive medications and supportive care interventions—such as corticosteroids, immunomodulators, and mechanical ventilation—have been examined in the therapy of sepsis [103]. The availability of cutting-edge supportive care technology, like extracorporeal membrane oxygenation and continuous renal replacement treatment, has increased the therapeutic options for sepsis patients in high-income nations [104]. The goal of the significant research into corticosteroids as an adjuvant medication in sepsis is to modulate the inflammatory response and enhance outcomes [105]. Nevertheless, there is still conflicting evidence supporting their routine usage in sepsis [106]. Low-dose corticosteroids are recommended by the Surviving Sepsis Campaign recommendations for patients in septic shock who continue to be hemodynamically unstable even after receiving sufficient fluid resuscitation and vasopressor treatment [96]. The guidelines do, however, recognize the weak quality of the available data and the possibility of negative consequences, including hyperglycemia and an elevated risk of infections [107]. Immunomodulatory treatments have been studied as possible adjuvant therapy in sepsis, including intravenous immunoglobulins, cytokine antagonists, and mesenchymal stem cells [108]. Nonetheless, there is still uncertainty regarding their effectiveness, and their usage is not usually advised in clinical settings [109]. Developing efficient immunomodulatory techniques is hampered by the variability of the sepsis population, the intricacy of the immunological response, and the timing of intervention [110]. For sepsis patients experiencing respiratory failure, mechanical ventilation is an essential supportive care strategy [111]. It has been demonstrated that patients with acute respiratory distress syndrome (ARDS) do better when lung-protective ventilation techniques, such as low tidal volumes and sufficient positive end-expiratory pressure (PEEP), are used in high-income nations [112]. In sepsis patients with ARDS, the Surviving Sepsis Campaign recommendations suggest aiming for a plateau pressure of less than 30 cm H_2_O and a tidal volume of 6 mL/kg of estimated body weight [96]. Access to sophisticated supportive care technology, like mechanical ventilation and renal replacement treatment, is frequently restricted in environments with minimal resources [113]. The provision of adequate supportive treatment to patients suffering from sepsis is significantly hampered by the absence of intensive care units, qualified staff, and essential equipment [114]. Basic therapies including oxygen therapy, hydration management, and infection control procedures are frequently the main focus in these settings [115]. Context-specific clinical recommendations, provider training, and healthcare infrastructure upgrading should be the main goals of strategies to increase access to and utilization of supportive care and complementary therapies in settings with low resources [33]. The gap in supportive care can be filled in part by using low-cost, portable devices like point-of-care ultrasound or non-invasive ventilation [116]. Further aiding in knowledge transfer and capacity building are collaborations with high-income universities and the creation of telemedicine networks (Table 2) [117].

## 7. What Is the Magnitude of the Economic Burden and Healthcare Costs across Continents?

Sepsis and septic shock impose a significant economic burden on healthcare systems, patients, and families worldwide. The costs associated with sepsis management vary across different regions and healthcare settings, with substantial direct and indirect costs that impact both developed and developing countries. The direct costs of sepsis and septic shock management include expenses related to hospitalization, intensive care unit (ICU) stays, medications, diagnostic tests, and medical procedures [118]. In high-income countries, the cost of sepsis care is substantial, with estimates ranging from $20,000 to $50,000 per patient episode [119,120]. In the United States, sepsis is one of the most expensive conditions treated in hospitals, accounting for over $20 billion in annual healthcare costs [11]. Similar high costs have been reported in Europe, with sepsis-related expenses ranging from €7500 to €27,000 per patient [118,121]. In low- and middle-income countries (LMICs), the direct costs of sepsis care are generally lower than in high-income countries, but they still represent a significant burden on healthcare systems and patients [43]. A study from Brazil estimated the average cost of sepsis treatment to be approximately $10,000 per patient, with higher costs associated with more severe disease [122]. In sub-Saharan Africa, the direct costs of sepsis care can be catastrophic for patients and families, often exceeding monthly household incomes [114,123]. The variability in direct costs across different healthcare systems can be attributed to factors such as the level of intensive care services, the availability of advanced medical technologies, and the use of expensive medications [124]. In resource-limited settings, the lack of access to essential medicines, diagnostic tools, and critical care facilities may result in lower direct costs but also poorer patient outcomes [125]. In addition to the direct costs, sepsis and septic shock also impose significant indirect costs on patients, families, and societies [126]. Indirect costs include lost productivity due to illness, disability, and premature mortality [127]. Sepsis survivors often experience long-term physical, cognitive, and psychological impairments that affect their ability to work and engage in daily activities [126,128]. These impairments can lead to reduced income, increased healthcare utilization, and diminished quality of life [129]. The long-term consequences of sepsis extend beyond the individual patient, affecting families and caregivers [130]. Family members may need to take time off work to provide care for sepsis survivors, leading to lost wages and economic strain. The emotional and psychological burden of caring for a sepsis survivor can also be substantial, with increased rates of depression, anxiety, and post-traumatic stress disorder among caregivers [131]. In LMICs, the indirect costs of sepsis can be particularly devastating for patients and families. The loss of income due to illness or death can push households into poverty, exacerbating existing economic inequalities [132]. The lack of social safety nets and limited access to healthcare services can further compound the economic burden of sepsis in these settings [133]. The high economic burden of sepsis and septic shock has significant implications for healthcare systems and resource allocation worldwide. In developed countries, the increasing incidence of sepsis and the growing demand for critical care services have put a strain on healthcare budgets [4]. Hospitals and healthcare systems must allocate substantial resources to manage sepsis patients, often at the expense of other healthcare priorities [134]. The aging population and the increasing prevalence of chronic comorbidities in developed countries are expected to further drive up the costs of sepsis care in the coming years [135]. In developing countries, the economic burden of sepsis poses significant challenges for already resource-constrained healthcare systems [123]. The high costs of sepsis care can divert limited resources away from other essential health services, such as maternal and child health, infectious disease control, and primary care [57]. The lack of access to adequate healthcare facilities, trained healthcare providers, and essential medicines in many LMICs can lead to delayed or inadequate sepsis care, resulting in higher mortality rates and greater economic losses [136]. Addressing the economic burden of sepsis requires a multifaceted approach that includes efforts to prevent infections, improve early recognition and treatment, and optimize resource allocation [43]. In developed countries, strategies such as the implementation of sepsis care bundles, the use of electronic health records to facilitate early detection, and the development of cost-effective diagnostic and therapeutic tools can help reduce the economic impact of sepsis [95,137]. In developing countries, strengthening healthcare systems, improving access to essential medicines and critical care services, and implementing cost-effective sepsis management protocols can help mitigate the economic burden of sepsis [138,139].

## 8. What Prevention Strategies and Quality Improvement Initiatives May Be Promoted Worldwide?

Global healthcare systems place a high premium on preventing sepsis and providing patients with sepsis with better care. To address these issues, a variety of approaches and programs have been put into place in various healthcare settings and geographical areas while taking into consideration regional contexts and available resources.

Sepsis must be detected early in order to provide prompt treatment and better patient outcomes. To aid in the early detection of sepsis, screening instruments and early warning systems have been created and tailored to various healthcare environments [140]. Electronic health records (EHRs) and automated alarm systems have been used in high-income nations to identify hospitalized patients who are at risk of sepsis [141]. These systems employ algorithms to set off alarms and facilitate early assessment and therapy based on vital signs, test results, and other clinical data [142]. Simplified screening techniques and tools have been created to help identify sepsis early in low- and middle-income countries (LMICs), where access to advanced technology and EHRs may be limited [139]. One method that has been suggested for use at the bedside in resource-constrained settings to identify patients at risk of sepsis is the “Quick Sepsis-related Organ Failure Assessment” (qSOFA) score [1]. The three-parameter (systolic blood pressure, respiratory rate, and mental state) qSOFA score has been validated in several LMIC populations and has demonstrated potential in identifying patients at increased risk of death [40,70]. In certain low- and middle-income countries (LMICs), community-based screening and early referral programs have been introduced to enhance the timely detection of sepsis, especially in isolated and rural regions [143]. Community health workers are frequently trained as part of these initiatives to identify sepsis symptoms and signs and to facilitate rapid referral to medical institutions [144]. For screening methods and protocols to be successfully used in various healthcare settings, they must be tailored to the local context, taking into consideration linguistic hurdles, cultural considerations, and resource availability [69]. Sepsis incidence can be decreased by the use of infection prevention and control (IPC) strategies, especially in hospital environments [145]. Fundamental IPC practices that have been extensively promoted in many different regions include hand hygiene, using personal protective equipment properly, and cleaning and disinfecting surroundings [146]. Comprehensive IPC programs have been introduced in high-income countries to minimize sepsis and healthcare-associated infections (HAIs) [147]. These programs include surveillance systems, outbreak investigation and treatment, and continual teaching and training of healthcare personnel. Implementing IPC in LMICs is fraught with difficulties due to a lack of funding, poor infrastructure, and conflicting healthcare objectives [132]. To improve IPC practices in resource-constrained situations, creative tactics like the “care bundle” approach and the application of alcohol-based hand rub have been modified [148,149]. With an emphasis on leadership, teaching, monitoring, and feedback, the World Health Organization (WHO) has created guidelines and tools to assist the implementation of IPC programs in low- and middle-income countries (LMICs) [150]. Another crucial element of preventing sepsis is the implementation of antimicrobial stewardship programs (ASPs), which optimize the use of antibiotics while limiting the emergence and spread of antibiotic resistance [151]. ASPs have been widely adopted in high-income nations using tactics such as formulary restriction, guideline creation, and prospective audit and feedback [152]. In order to direct antibiotic therapy and minimize needless use, fast diagnostic tests and biomarkers have also been added to ASPs [153]. Implementing ASPs in LMICs is hampered by issues such as insufficient capacity for diagnosis, insufficient control over the sale of antibiotics, and a shortage of workers with the necessary training [87]. Several solutions have been suggested to address these issues, including tailoring ASP tactics to the local environment, emphasizing education, and making use of current networks and resources [154]. In order to facilitate the creation and application of ASPs in LMICs, the Global Antibiotic Resistance Partnership (GARP) was founded, encouraging a multidisciplinary and context-specific strategy [155]. Initiatives aimed at improving quality and preventing sepsis must prioritize education and raising public awareness. To enhance the diagnosis and treatment of sepsis, educational initiatives aimed at medical professionals have been put in place in high-income nations [39]. To improve knowledge and abilities, these programs frequently include multidisciplinary approaches, case-based learning, and simulation training [156]. To increase public awareness of sepsis and encourage early care-seeking behavior, public awareness programs have been created, such as the “Surviving Sepsis Campaign” and “World Sepsis Day” [39,157]. Limited resources, language and cultural hurdles, and conflicting health objectives are some of the particular difficulties faced by educational programs and public awareness campaigns in low- and middle-income countries (LMICs) [113]. The success of educational initiatives depends on their customization to the local environment, use of culturally relevant materials and messaging, and involvement of stakeholders and community leaders [8,158]. In environments with limited resources, using peer educators and community health professionals has been suggested as a tactic to encourage behavior change and spread knowledge [33]. Innovative strategies to improve sepsis education and awareness in LMICs have also been investigated [159]. These strategies include the use of social media platforms and mobile health (mHealth) technology. For instance, the Global Sepsis Alliance’s “Stop Sepsis” mobile application offers teaching tools and information for healthcare professionals as well as the general public, tailored to various languages and situations. In environments with limited resources, utilizing pre-existing community networks and working with nearby organizations and media sources can also help increase the reach and effect of campaigns raising awareness of sepsis [144]. Programs for quality improvement (QI) and care bundles have been widely used to improve patient outcomes and raise the standard of sepsis care. Evidence-based protocols and care packages have been utilized in high-income countries as part of QI programs aimed at early detection and treatment of sepsis [74]. A number of care packages that have been produced by the “Surviving Sepsis Campaign”—such as prompt antibiotic administration, blood culture collection, and early lactate measurement—have been linked to lower mortality rates and better adherence to sepsis recommendations [95,96]. QI initiatives in LMICs have been tailored to the local environment, taking into consideration the limitations of the healthcare system and the resources that are available [39]. The “WHO Global Maternal Sepsis Study” showed that a QI bundle with an emphasis on early detection, prompt fluid resuscitation, and antibiotic therapy could be successfully implemented for the care of maternal sepsis in 52 LMICs [42]. A sepsis treatment bundle tailored to the needs of low- and middle-income countries (LMICs) has also been established by the “Sepsis in Resource-Limited Nations” (SiREN) project, with an emphasis on the significance of early detection, suitable antibiotic therapy, and source control [159]. Several nations have investigated using clinical decision support systems and electronic health records as a means of enhancing the execution of QI projects and sepsis treatment packages [160]. Telemedicine and mobile health technologies have been suggested as ways to help LMICs, especially in rural and isolated areas, conduct sepsis quality improvement programs [161]. The effective execution and long-term viability of sepsis quality improvement (QI) programs in various healthcare settings depend on involving local stakeholders, such as administrators, policymakers, and healthcare professionals [159].

## 9. What Are Challenges and Future Directions in Global Sepsis Management?

**Figure 2:**
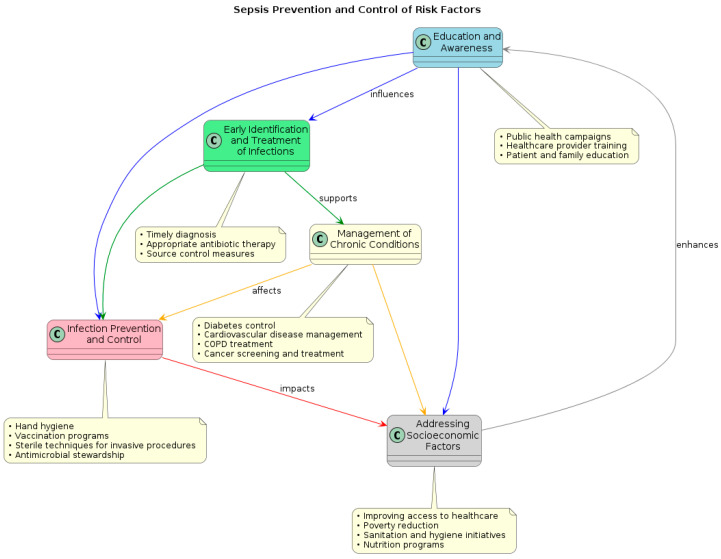
Flow diagram of prevention policies and control of risk factors for sepsis and septic shock.

Sepsis remains a serious worldwide health concern despite tremendous breakthroughs in our understanding of its etiology and therapy. Global sepsis burden management necessitates a multimodal strategy that considers the particular opportunities and challenges in many geographic locations and healthcare contexts (Figure 2). 

The absence of uniform diagnostic standards and treatment strategies across various geographic locations and healthcare environments is one of the main obstacles in the management of sepsis [162]. It has been questioned whether the current definition of sepsis, which is based on the Sepsis-3 criteria, is applicable to low- and middle-income countries (LMICs) because it was established and validated predominantly in high-income countries [157]. The precise identification of sepsis based on these criteria may be hampered by the restricted availability of laboratory tests and imaging modalities in resource-constrained settings [5]. Furthermore, depending on local infectious disease epidemiology, healthcare infrastructure, and resource availability, therapeutic approaches to sepsis therapy may differ significantly between areas [8]. In settings with low resources, the guidelines of the Surviving Sepsis Campaign, which have gained widespread adoption in affluent nations, might not be suitable or possible [125]. Improving sepsis outcomes in low- and middle-income countries (LMICs) requires tailoring these recommendations to the local environment, taking into consideration resource availability and the particular difficulties faced by healthcare providers [96]. Future work should concentrate on creating and evaluating sepsis management algorithms and context-specific diagnostic criteria that take into consideration the potential and constraints of various healthcare settings [160]. This can entail the application of streamlined clinical scores, like the fast Sequential Organ Failure Assessment (qSOFA) score, which is simple to use at the patient’s bedside and does not require laboratory testing [163]. Developing point-of-care diagnostic assays is another promising way to improve sepsis management in places with limited resources, as they can quickly identify the underlying organism and provide tailored antibiotic therapy [1]. One method that has shown promise for enhancing sepsis diagnosis, prognosis, and treatment is the use of biomarkers [164]. Procalcitonin, C-reactive protein, and lactate are a few examples of biomarkers that have been thoroughly investigated in sepsis and have demonstrated promise for directing antibiotic therapy and forecasting results [165]. However, depending on variables including genetic background, comorbidities, and infectious disease epidemiology, these biomarkers’ performance may change in various populations and healthcare settings [166]. Sepsis results may be improved by the application of precision medicine, which focuses on individualized treatment plans based on a patient’s genetic, clinical, and environmental factors [167]. It has been determined that certain genetic polymorphisms may affect a person’s vulnerability to sepsis and reaction to treatment [168]. Research is currently being conducted on the application of pharmacogenomic techniques to direct the choice and dosage of antimicrobial medicines according to a person’s genetic profile [63]. Precision medicine approaches to sepsis care in low- and middle-income countries (LMICs) are confronted with various obstacles, such as restricted genetic testing availability and a dearth of proven pharmacogenomic algorithms in varied populations [169]. Subsequent investigations ought to concentrate on detecting biomarkers and genetic variations unique to a community, which might direct the treatment of sepsis in various geographical locations and medical environments [170]. Developing accessible, point-of-care genetic testing platforms and incorporating pharmacogenomic data into clinical decision support systems could serve as a means of addressing these obstacles and enhancing sepsis outcomes in environments with limited resources [64]. Few significant advances have been made in the creation of novel treatment medicines for sepsis in spite of decades of research [171]. Many immunomodulatory drugs, including IL-1 receptor antagonists and anti-TNF antibodies, have failed in human trials, which has caused a reevaluation of the existing strategies for developing drugs for sepsis [172]. Novel treatment targets, including the endothelium, coagulation cascade, and microbiota, have been the focus of recent study and may be important players in the pathophysiology of sepsis [173]. The particular opportunities and challenges found in various hospital settings should be considered in the development of novel treatment strategies for sepsis [174]. Another interesting strategy for sepsis medication development is the repurposing of already-approved medications, such as beta-blockers and statins, which have pleiotropic effects on the immune system and cardiovascular function [175]. Hemoadsorption and hemofiltration are two examples of extracorporeal blood purification methods that have come to light as possible sepsis treatment approaches [176]. By removing inflammatory mediators and toxins from the bloodstream, these methods hope to reduce organ failure and the systemic inflammatory response that accompany sepsis [177]. It is still unknown, nevertheless, whether these therapies are cost-effective and clinically effective in various hospital contexts [104]. Subsequent investigations ought to concentrate on pinpointing innovative treatment objectives and strategies that are suitable for a range of patient demographics and medical environments [178]. Priority should be given to the creation of accessible, affordable therapies that are simple to use in environments with limited resources [179]. The development of innovative sepsis treatments may be sped up by the application of adaptive clinical trial designs, which enable the quick identification of promising medications and the discontinuation of ineffective ones [180]. A coordinated, multidisciplinary strategy involving cooperation between physicians, researchers, legislators, and public health specialists from various geographic locations and healthcare settings is needed to address the global burden of sepsis [181]. The creation of international research networks, such the International Severe Sepsis and Septic Shock Collaborative Group and the Global Sepsis Alliance, has made it easier for sepsis specialists all over the world to share best practices and expertise [182]. Nonetheless, notable discrepancies persist in funding and research output related to sepsis between high-income and low- and middle-income nations [183]. Since most sepsis research is done in wealthy nations, its conclusions could not apply directly to situations when resources are scarce [184]. The inclusion of LMIC sites and the creation of research questions and procedures tailored to the specific environment should be given top priority in future projects [4]. Promoting international cooperation and research projects to address the global sepsis burden should incorporate a number of crucial tactics. First, creating research collaborations between high-income and LMIC universities can support the development of research knowledge and capacity in environments with limited resources [185]. Second, the creation of uniform instruments for data gathering and reporting can make it easier to compare and combine data from various research and geographical areas [186]. Third, in order to guarantee the applicability and acceptability of study findings, local stakeholders such as legislators, healthcare professionals, and community members must be involved [157]. Ultimately, in order to lower the worldwide sepsis burden, research findings must be translated into clinical practice and public health policy [7]. This calls for the creation of procedures and guidelines that are appropriate for the given context, the bolstering of healthcare infrastructure and systems, and the public’s and healthcare providers’ empowerment and education [41]. Political will and funding for sepsis prevention and control can be mobilized by including sepsis management into larger global health programs like the Sustainable Development Goals and Universal Health Coverage [187].

## 10. Conclusions

Millions of individuals worldwide suffer from sepsis and septic shock, which continue to be major global health burdens that result in high rates of morbidity, mortality, and medical expenses. The differences in sepsis epidemiology, risk factors, treatment, and outcomes between countries and healthcare environments have been brought to light by this thorough review. Sepsis varies greatly in incidence and fatality rates between geographical areas, with low- and middle-income countries (LMICs) experiencing a disproportionately high illness burden. Sepsis incidence and mortality are higher in these settings due to a number of factors, including a high frequency of infectious illnesses, restricted access to important medications and diagnostic equipment, and poor healthcare infrastructure. The rising incidence of chronic illnesses, an aging population, and the advent of microorganisms resistant to antibiotics are the main causes of the substantial sepsis burden in high-income nations. The review has also brought to light the differences in risk factors and comorbidities between continents. The incidence and prognosis of sepsis are significantly influenced by a number of factors, including environmental and socioeconomic factors, chronic health conditions, acute medical and surgical conditions, and demographic factors. Population-specific variations in genetic variables and sepsis susceptibility highlight the need for context-specific research and therapies. Healthcare environments vary in how they handle sepsis and septic shock. Those with inadequate resources have a difficult time identifying, diagnosing, and treating sepsis in a timely manner. The scarcity of diagnostic instruments, antimicrobial agents, and supportive care technologies in these environments causes treatment to be delayed or insufficient, which raises mortality rates. The application of evidence-based guidelines and care bundles has improved outcomes in high-income nations, but there is still room for improvement, especially in the treatment of organ dysfunction related to sepsis and the long-term effects of sepsis. The public, politicians, researchers, and healthcare professionals must work together to address the global burden of septic shock and sepsis.

## Figures and Tables

**Table 1:** Epidemiology of sepsis and septic shock.

Study	Country/Region	Period	Population	Key Findings
Fleischmann et al. (2016) [4]	Global	1990–2017	Hospital-treated patients	- Global incidence of hospital-treated sepsis: 31.5 million cases per year - Overall mortality: 17% - Sepsis incidence and mortality varied substantially across regions, with the highest burden in low- and middle-income countries (LMICs)
Rudd et al. (2020) [5]	Global	2017	General population	- Global age-standardized sepsis-related mortality rate: 148.1 deaths per 100,000 population - 85% of sepsis cases and 84% of sepsis-related deaths occurred in LMICs - Highest mortality rates observed in sub-Saharan Africa, Oceania, and South Asia
Angus et al. (2001) [43]	United States	1995	Hospital-treated patients	- Incidence of severe sepsis: 300 cases per 100,000 population - Mortality: 28.6% - Annual cost of sepsis care: $16.7 billion - Incidence and mortality increased with age, with the highest rates among individuals aged 65 years and older
Ahmed et al. (2021) [25]	Pakistan	2013–2014	Adult patients with sepsis or septic shock	- Prevalence of sepsis: 61.25% - Prevalence of severe sepsis or septic shock: 38.75% - ICU admission: 10% - Overall mortality of sepsis: 9.8% - Overall mortality of sepsis shock: 22.8%
Weng et al. (2023) [24]	China	2017–2019	Hospitalized patients with sepsis	- Incidence of sepsis: 422 cases per 100,000 in 2019 - Incidence in neonates: 8.7% - Incidence in children: 11.7% - Incidence in adults: 57.5%

**Table 2:** Management of sepsis and septic shock in different healthcare settings.

Study	Country/Region	Population	Study Design	Key Findings	Implications
Rhodes et al. (2017) [34]	International	Adult patients with sepsis and septic shock	Evidence-based guideline	- Recommendations for diagnosis, resuscitation, antimicrobial therapy, source control, and supportive care - Emphasis on early recognition and timely intervention - Guidance on hemodynamic support, ventilation, and adjunctive therapies	- Provides a framework for standardized sepsis management across different healthcare settings - Highlights the importance of a multidisciplinary approach and continuous quality improvement
Schultz et al. (2017) [8]	Low- and middle-income countries (LMICs)	Patients with sepsis and septic shock	Review article	- Challenges in sepsis management in resource-limited settings, including lack of skilled staff, inadequate infrastructure, and limited access to essential therapies - Suggestions for improving care, such as training healthcare workers, developing context-specific protocols, and enhancing access to resources	- Emphasizes the need for tailored interventions and strategies to address the unique challenges faced by LMICs - Underscores the importance of global collaboration and knowledge sharing to improve sepsis outcomes in resource-limited settings
Levy et al. (2018) [90]	International	Adult patients with sepsis and septic shock	Guideline update	- Update of the Surviving Sepsis Campaign bundle - Focus on early recognition, prompt resuscitation, antimicrobial therapy, and source control - Introduction of the hour-1 bundle, emphasizing the critical importance of early interventions	- Provides an updated framework for sepsis management based on the latest evidence - Stresses the importance of timely and coordinated care to improve sepsis outcomes - Underscores the need for continuous education and performance improvement initiatives to ensure adherence to best practices

## Data Availability

Not applicable.

## References

[1] Singer M., Deutschman C.S., Seymour C.W., Shankar-Hari M., Annane D., Bauer M., Bellomo R., Bernard G.R., Chiche J.D., Coopersmith C.M. (2016). The third international consensus definitions for sepsis and septic shock (Sepsis-3). JAMA.

[2] Hotchkiss R.S., Moldawer L.L., Opal S.M., Reinhart K., Turnbull I.R., Vincent J.-L. (2016). Sepsis and septic shock. Nat. Rev. Dis. Primer.

[3] Shankar-Hari M., Phillips G.S., Levy M.L., Seymour C.W., Liu V.X., Deutschman C.S., Angus D.C., Rubenfeld G.D., Singer M., Sepsis Definitions Task Force (2016). Developing a New Definition and Assessing New Clinical Criteria for Septic Shock: For the Third International Consensus Definitions for Sepsis and Septic Shock (Sepsis-3). JAMA.

[4] Fleischmann C., Scherag A., Adhikari N.K., Hartog C.S., Tsaganos T., Schlattmann P., Angus D.C., Reinhart K., International Forum of Acute Care Trialists (2016). Assessment of Global Incidence and Mortality of Hospital-treated Sepsis. Current Estimates and Limitations. Am. J. Respir. Crit. Care Med..

[5] Rudd K.E., Johnson S.C., Agesa K.M., Shackelford K.A., Tsoi D., Kievlan D.R., Colombara D.V., Ikuta K.S., Kissoon N., Finfer S. (2020). Global, regional, and national sepsis incidence and mortality, 1990-2017: Analysis for the Global Burden of Disease Study. Lancet.

[6] WHO Calls for Global Action on Sepsis—Cause of 1 in 5 Deaths Worldwide. Consultato: 11 Giugno 2024. https://www.who.int/news/item/08-09-2020-who-calls-for-global-action-on-sepsis---cause-of-1-in-5-deaths-worldwide.

[7] Adhikari N.K.J., Fowler R.A., Bhagwanjee S., Rubenfeld G.D. (2010). Critical care and the global burden of critical illness in adults. Lancet.

[8] Schultz M.J., Dunser M.W., Dondorp A.M., Adhikari N.K.J., Iyer S., Kwizera A., Lubell Y., Papali A., Pisani L., Riviello B.D. (2017). Current challenges in the management of sepsis in ICUs in resource-poor settings and suggestions for the future. Intensive Care Med..

[9] Martin G.S., Mannino D.M., Eaton S., Moss M. (2003). The epidemiology of sepsis in the United States from 1979 through 2000. N. Engl. J. Med..

[10] Prescott H.C., Angus D.C. (2018). Enhancing Recovery From Sepsis: A Review. JAMA.

[11] Torio C.M., Moore B.J. (2006). National Inpatient Hospital Costs: The Most Expensive Conditions by Payer, 2013. Healthcare Cost and Utilization Project (HCUP) Statistical Briefs.

[12] Martin G.S., Mannino D.M., Moss M. (2006). The effect of age on the development and outcome of adult sepsis. Crit. Care Med..

[13] Cohen J., Vincent J.L., Adhikari N.K., Machado F.R., Angus D.C., Calandra T., Jaton K., Giulieri S., Delaloye J., Opal S. (2015). Sepsis: A roadmap for future research. Lancet Infect. Dis..

[14] Wells G.A., Shea B., O’Connell D., Peterson J., Welch V., Losos M., Tugwell P. (2014). The Newcastle-Ottawa Scale (NOS) for Assessing the Quality of Nonrandomised Studies in Meta-Analyses. https://www.ohri.ca/programs/clinical_epidemiology/oxford.asp.

[15] Bone R.C., Balk R.A., Cerra F.B., Dellinger R.P., Fein A.M., Knaus W.A., Schein R.M., Sibbald W.J., Abrams J.H., Bernard G.R. (1992). Definitions for sepsis and organ failure and guidelines for the use of innovative therapies in sepsis. The ACCP/SCCM Consensus Conference Committee. American College of Chest Physicians/Society of Critical Care Medicine. Chest.

[16] Vincent J.L. (1997). Dear SIRS, I’m sorry to say that I don’t like you. Crit. Care Med..

[17] Levy M.M., Fink M.P., Marshall J.C., Abraham E., Angus D., Cook D., Cohen J., Opal S.M., Vincent J.L., Ramsay G. (2003). 2001 SCCM/ESICM/ACCP/ATS/SIS International Sepsis Definitions Conference. Crit. Care Med..

[18] Fleischmann-Struzek C., Mellhammar L., Rose N., Cassini A., Rudd K.E., Schlattmann P., Allegranzi B., Reinhart K. (2020). Incidence and mortality of hospital- and ICU-treated sepsis: Results from an updated and expanded systematic review and meta-analysis. Intensive Care Med..

[19] Rhee C., Dantes R., Epstein L., Murphy D.J., Seymour C.W., Iwashyna T.J., Kadri S.S., Angus D.C., Danner R.L., Fiore A.E. (2017). Incidence and Trends of Sepsis in US Hospitals Using Clinical vs Claims Data, 2009–2014. JAMA.

[20] Jolley R.J., Quan H., Jetté N., Sawka K.J., Diep L., Goliath J., Roberts D.J., Yipp B.G., Doig C.J. (2015). Validation and optimisation of an ICD-10-coded case definition for sepsis using administrative health data. BMJ Open.

[21] Padkin A., Goldfrad C., Brady A.R., Young D., Black N., Rowan K. (2003). Epidemiology of severe sepsis occurring in the first 24 hrs in intensive care units in England, Wales, and Northern Ireland. Crit. Care Med..

[22] Fleischmann-Struzek C., Mikolajetz A., Schwarzkopf D., Cohen J., Hartog C.S., Pletz M., Gastmeier P., Reinhart K. (2018). Challenges in assessing the burden of sepsis and understanding the inequalities of sepsis outcomes between National Health Systems: Secular trends in sepsis and infection incidence and mortality in Germany. Intensive Care Med..

[23] Southeast Asia Infectious Disease Clinical Research Network (2017). Causes and outcomes of sepsis in southeast Asia: A multinational multicentre cross-sectional study. Lancet Glob. Health.

[24] Weng L., Xu Y., Yin P., Wang Y., Chen Y., Liu W., Li S., Peng J.-M., Dong R., Hu X.-Y. (2023). National incidence and mortality of hospitalized sepsis in China. Crit. Care.

[25] Ahmed A.S., Hussain E., Haleem S., Ahmed N., Latif A. (2021). Epidemiology of sepsis, based on ICD-9 coding, a tertiary care experience from Pakistan. Trends Anaesth. Crit. Care.

[26] Liu V., Escobar G.J., Greene J.D., Soule J., Whippy A., Angus D.C., Iwashyna T.J. (2014). Hospital deaths in patients with sepsis from 2 independent cohorts. JAMA.

[27] Shankar-Hari M., Harrison D.A., Rowan K.M. (2016). Differences in Impact of Definitional Elements on Mortality Precludes International Comparisons of Sepsis Epidemiology—A Cohort Study Illustrating the Need for Standardized Reporting. Crit. Care Med..

[28] Machado F.R., Cavalcanti A.B., Bozza F.A., Ferreira E.M., Angotti Carrara F.S., Sousa J.L., Caixeta N., Salomao R., Angus D.C., Pontes Azevedo L.C. (2017). The epidemiology of sepsis in Brazilian intensive care units (the Sepsis PREvalence Assessment Database, SPREAD): An observational study. Lancet Infect. Dis..

[29] Divatia J.V., Amin P.R., Ramakrishnan N., Kapadia F.N., Todi S., Sahu S., Govil D., Chawla R., Kulkarni A.P., Samavedam S. (2016). Intensive Care in India: The Indian Intensive Care Case Mix and Practice Patterns Study. Indian J. Crit. Care Med. Peer-Rev. Off. Publ. Indian Soc. Crit. Care Med..

[30] Kaukonen K.-M., Bailey M., Suzuki S., Pilcher D., Bellomo R. (2014). Mortality related to severe sepsis and septic shock among critically ill patients in Australia and New Zealand, 2000-2012. JAMA.

[31] Levy M.M., Rhodes A., Phillips G.S., Townsend S.R., Schorr C.A., Beale R., Osborn T., Lemeshow S., Chiche J.D., Artigas A. (2014). Surviving Sepsis Campaign: Association between performance metrics and outcomes in a 7.5-year study. Intensive Care Med..

[32] Ferrer R., Martin-Loeches I., Phillips G., Osborn T.M., Townsend S., Dellinger R.P., Artigas A., Schorr C., Levy M.M. (2014). Empiric antibiotic treatment reduces mortality in severe sepsis and septic shock from the first hour: Results from a guideline-based performance improvement program. Crit. Care Med..

[33] Thwaites C.L., Lundeg G., Dondorp A.M., Sepsis in Resource-Limited Settings–Expert Consensus Recommendations Group of the European Society of Intensive Care Medicine (ESICM) and the Mahidol-Oxford Research Unit (MORU) in Bangkok, Thailand (2016). Recommendations for infection management in patients with sepsis and septic shock in resource-limited settings. Intensive Care Med..

[34] Esper A.M., Moss M., Lewis C.A., Nisbet R., Mannino D.M., Martin G.S. (2006). The role of infection and comorbidity: Factors that influence disparities in sepsis. Crit. Care Med..

[35] Esteban A., Frutos-Vivar F., Ferguson N.D., Peñuelas O., Lorente J.A., Gordo F., Honrubia T., Algora A., Bustos A., García G. (2007). Sepsis incidence and outcome: Contrasting the intensive care unit with the hospital ward. Crit. Care Med..

[36] Dondorp A.M., Dünser M.W., Schultz M.J. (2019). Sepsis Management in Resource-limited Settings.

[37] Ranjit S., Kissoon N., Jayakumar I. (2005). Aggressive management of dengue shock syndrome may decrease mortality rate: A suggested protocol. Pediatr. Crit. Care Med. J. Soc. Crit. Care Med. World Fed. Pediatr. Intensive Crit. Care Soc..

[38] Jacob S.T., Moore C.C., Banura P., Pinkerton R., Meya D., Opendi P., Reynolds S.J., Kenya-Mugisha N., Mayanja-Kizza H., Scheld W.M. (2009). Severe sepsis in two Ugandan hospitals: A prospective observational study of management and outcomes in a predominantly HIV-1 infected population. PLoS ONE.

[39] Rhodes A., Evans L.E., Alhazzani W., Levy M.M., Antonelli M., Ferrer R., Kumar A., Sevransky J.E., Sprung C.L., Nunnally M.E. (2017). Surviving Sepsis Campaign: International Guidelines for Management of Sepsis and Septic Shock: 2016. Intensive Care Med..

[40] Huson M.A.M., Kalkman R., Grobusch M.P., van der Poll T. (2017). Predictive value of the qSOFA score in patients with suspected infection in a resource limited setting in Gabon. Travel Med. Infect. Dis..

[41] Maitland K., Kiguli S., Opoka R.O., Engoru C., Olupot-Olupot P., Akech S.O., Nyeko R., Mtove G., Reyburn H., Lang T. (2011). Mortality after fluid bolus in African children with severe infection. N. Engl. J. Med..

[42] Andrews B., Semler M.W., Muchemwa L., Kelly P., Lakhi S., Heimburger D.C., Mabula C., Bwalya M., Bernard G.R. (2017). Effect of an Early Resuscitation Protocol on In-hospital Mortality Among Adults With Sepsis and Hypotension: A Randomized Clinical Trial. JAMA.

[43] Angus D.C., Linde-Zwirble W.T., Lidicker J., Clermont G., Carcillo J., Pinsky M.R. (2001). Epidemiology of severe sepsis in the United States: Analysis of incidence, outcome, and associated costs of care. Crit. Care Med..

[44] Angus D.C., Kelley M.A., Schmitz R.J., White A., Popovich J., Committee on Manpower for Pulmonary and Critical Care Societies (COMPACCS), Caring for the Critically Ill Patient (2000). Current and projected workforce requirements for care of the critically ill and patients with pulmonary disease: Can we meet the requirements of an aging population?. JAMA.

[45] Adrie C., Azoulay E., Francais A., Clec’h C., Darques L., Schwebel C., Nakache D., Jamali S., Goldgran-Toledano D., Garrouste-Orgeas M. (2007). Influence of gender on the outcome of severe sepsis: A reappraisal. Chest.

[46] Sakr Y., Elia C., Mascia L., Barberis B., Cardellino S., Livigni S., Fiore G., Filippini C., Ranieri V.M. (2013). The influence of gender on the epidemiology of and outcome from severe sepsis. Crit. Care.

[47] Esper A.M., Moss M., Martin G.S. (2009). The effect of diabetes mellitus on organ dysfunction with sepsis: An epidemiological study. Crit. Care.

[48] Wang H.E., Shapiro N.I., Griffin R., Safford M.M., Judd S., Howard G. (2012). Chronic medical conditions and risk of sepsis. PLoS ONE.

[49] Danai P.A., Moss M., Mannino D.M., Martin G.S. (2006). The epidemiology of sepsis in patients with malignancy. Chest.

[50] Yende S., Alvarez K., Loehr L., Folsom A.R., Newman A.B., Weissfeld L.A., Wunderink R.G., Kritchevsky S.B., Mukamal K.J., London S.J. (2013). Epidemiology and long-term clinical and biologic risk factors for pneumonia in community-dwelling older Americans: Analysis of three cohorts. Chest.

[51] Yende S., Iwashyna T.J., Angus D.C. (2014). Interplay between sepsis and chronic health. Trends Mol. Med..

[52] Vincent J.-L., Sakr Y., Sprung C.L., Ranieri V.M., Reinhart K., Gerlach H., Moreno R., Carlet J., Le Gall J.-R., Payen D. (2006). Sepsis in European intensive care units: Results of the SOAP study. Crit. Care Med..

[53] Leligdowicz A., Dodek P.M., Norena M., Wong H., Kumar A., Kumar A. (2014). Co-operative Antimicrobial Therapy of Septic Shock Database Research Group. Association between source of infection and hospital mortality in patients who have septic shock. Am. J. Respir. Crit. Care Med..

[54] Moore L.J., Moore F.A., Todd S.R., Jones S.L., Turner K.L., Bass B.L. (2010). Sepsis in general surgery: The 2005-2007 national surgical quality improvement program perspective. Arch. Surg. Chic. Ill 1960.

[55] Greenhalgh D.G., Saffle J.R., Holmes J.H., Gamelli R.L., Palmieri T.L., Horton J.W., Tompkins R.G., Traber D.L., Mozingo D.W., Deitch E.A. (2007). American Burn Association consensus conference to define sepsis and infection in burns. J. Burn Care Res. Off. Publ. Am. Burn. Assoc..

[56] Osborn T.M., Tracy J.K., Dunne J.R., Pasquale M., Napolitano L.M. (2004). Epidemiology of sepsis in patients with traumatic injury. Crit. Care Med..

[57] Cheng A.C., West T.E., Limmathurotsakul D., Peacock S.J. (2008). Strategies to reduce mortality from bacterial sepsis in adults in developing countries. PLoS Med..

[58] Becker J.U., Theodosis C., Jacob S.T., Wira C.R., Groce N.E. (2009). Surviving sepsis in low-income and middle-income countries: New directions for care and research. Lancet Infect. Dis..

[59] Golding N., Burstein R., Longbottom J., Browne A.J., Fullman N., Osgood-Zimmerman A., Earl L., Bhatt S., Cameron E., Casey D.C. (2017). Mapping under-5 and neonatal mortality in Africa, 2000–2015: A baseline analysis for the Sustainable Development Goals. Lancet.

[60] Goodwin A.J., Nadig N.R., McElligott J.T., Simpson K.N., Ford D.W. (2016). Where You Live Matters: The Impact of Place of Residence on Severe Sepsis Incidence and Mortality. Chest.

[61] Rush B., Wiskar K., Celi L.A., Walley K.R., Russell J.A., McDermid R.C., Boyd J.H. (2018). Association of Household Income Level and In-Hospital Mortality in Patients With Sepsis: A Nationwide Retrospective Cohort Analysis. J. Intensive Care Med..

[62] Sutherland A.M., Walley K.R. (2009). Bench-to-bedside review: Association of genetic variation with sepsis. Crit. Care.

[63] Rautanen A., Mills T.C., Gordon A.C., Hutton P., Steffens M., Nuamah R., Chiche J.-D., Parks T., Chapman S.J., Davenport E.E. (2015). Genome-wide association study of survival from sepsis due to pneumonia: An observational cohort study. Lancet Respir. Med..

[64] Scherag A., Schöneweck F., Kesselmeier M., Taudien S., Platzer M., Felder M., Sponholz C., Rautanen A., Hill A.V.S., Hinds C.J. (2016). Genetic Factors of the Disease Course after Sepsis: A Genome-Wide Study for 28Day Mortality. EBioMedicine.

[65] Phillips K.L., Chiriboga D.A., Jang Y. (2012). Satisfaction with care: The role of patient-provider racial/ethnic concordance and interpersonal sensitivity. J. Aging Health.

[66] Dondorp A.M., Limmathurotsakul D., Ashley E.A. (2018). What’s wrong in the control of antimicrobial resistance in critically ill patients from low- and middle-income countries?. Intensive Care Med..

[67] Penno E.C., Baird S.J., Crump J.A. (2015). Cost-Effectiveness of Surveillance for Bloodstream Infections for Sepsis Management in Low-Resource Settings. Am. J. TroMed. Hyg..

[68] Lubell Y., Blacksell S.D., Dunachie S., Tanganuchitcharnchai A., Althaus T., Watthanaworawit W., Paris D.H., Mayxay M., Peto T.J., Dondorp A.M. (2015). Performance of C-reactive protein and procalcitonin to distinguish viral from bacterial and malarial causes of fever in Southeast Asia. BMC Infect. Dis..

[69] Huson M.A.M., Katete C., Chunda L., Ngoma J., Wallrauch C., Heller T., van der Poll T., Grobusch M.P. (2017). Application of the qSOFA score to predict mortality in patients with suspected infection in a resource-limited setting in Malawi. Infection.

[70] Rudd K.E., Johnson S.C., Agesa K.M., Shackelford K.A., Tsoi D., Kievlan D.R., Colombara D.V., Ikuta K.S., Kissoon N., Finfer S. (2018). Association of the Quick Sequential (Sepsis-Related) Organ Failure Assessment (qSOFA) Score With Excess Hospital Mortality in Adults With Suspected Infection in Low- and Middle-Income Countries. JAMA.

[71] Aluisio A.R., Garbern S., Wiskel T., Mutabazi Z.A., Umuhire O., Mbanjumucyo G., Mold J., Patel A., Shyaka E., Tuyisenge L. (2018). Mortality outcomes based on ED qSOFA score and HIV status in a developing low income country. Am. J. Emerg. Med..

[72] Jacob S.T., Lim M., Banura P., Bhagwanjee S., Bion J., Cheng A.C., Cohen H., Farrar J., Gove S., Hopewell P. (2013). Integrating sepsis management recommendations into clinical care guidelines for district hospitals in resource-limited settings: The necessity to augment new guidelines with future research. BMC Med..

[73] Andrews B., Muchemwa L., Kelly P., Lakhi S., Heimburger D.C., Bernard G.R. (2014). Simplified severe sepsis protocol: A randomized controlled trial of modified early goal-directed therapy in Zambia. Crit. Care Med..

[74] Kwizera A., Festic E., Dünser M.W. (2016). What’s new in sepsis recognition in resource-limited settings?. Intensive Care Med..

[75] Urayeneza O., Mujyarugamba P., Rukemba Z., Nyiringabo V., Ntihinyurwa P., Baelani J.I., Kwizera A., Bagenda D., Mer M., Hoffman M. (2018). Increasing Evidence-Based Interventions in Patients with Acute Infections in a Resource-Limited Setting: A Before-and-After Feasibility Trial in Gitwe, Rwanda. Crit. Care Med..

[76] Eze P., Idemili C.J., Lawani L.O. (2024). Evaluating health systems’ efficiency towards universal health coverage: A data envelopment analysis. Inq. J. Med. Care Organ. Provis. Financ..

[77] Kazungu J.S., Barasa E.W. (2017). Examining levels, distribution and correlates of health insurance coverage in Kenya. Trop. Med. Int. Health.

[78] Fadare J.O., Ogunleye O., Iliyasu G., Adeoti A., Schellack N., Engler D., Massele A., Godman B. (2019). Status of antimicrobial stewardship programmes in Nigerian tertiary healthcare facilities: Findings and implications. J. Glob. Antimicrob. Resist..

[79] Hadi U., Duerink D.O., Lestari E.S., Nagelkerke N.J., Werter S., Keuter M., Suwandojo E., Rahardjo E., van den Broek P., Gyssens I.C. (2008). Audit of antibiotic prescribing in two governmental teaching hospitals in Indonesia. Clin. Microbiol. Infect. Off. Publ. Eur. Soc. Clin. Microbiol. Infect. Dis..

[80] Ouedraogo A.S., Pierre H.J., Bañuls A.L., Ouédraogo R., Godreuil S. (2017). Emergence and spread of antibiotic resistance in West Africa: Contributing factors and threat assessment. Med. Sante Trop..

[81] Opintan J.A., Newman M.J., Arhin R.E., Donkor E.S., Gyansa-Lutterodt M., Mills-Pappoe W. (2015). Laboratory-based nationwide surveillance of antimicrobial resistance in Ghana. Infect. Drug Resist..

[82] Vernet G., Mary C., Altmann D.M., Doumbo O., Morpeth S., Bhutta Z.A., Klugman K.P., Roca A., Schito M., Zumla A. (2014). Surveillance for antimicrobial drug resistance in under-resourced countries. Emerg. Infect. Dis..

[83] Marshall J.C., Maier R.V., Jimenez M., Dellinger E.P. (2004). Source control in the management of severe sepsis and septic shock: An evidence-based review. Crit. Care Med..

[84] Mock C., Visser L., Denno D., Maier R. (1995). Aggressive fluid resuscitation and broad spectrum antibiotics decrease mortality from typhoid ileal perforation. Trop. Doctor..

[85] Shrime M.G., Dare A.J., Alkire B.C., O’Neill K., Meara J.G. (2015). Catastrophic expenditure to pay for surgery worldwide: A modelling study. Lancet Glob. Health.

[86] Weiser T.G., Regenbogen S.E., Thompson K.D., Haynes A.B., Lipsitz S.R., Berry W.R., Gawande A.A., Ozgediz D., Meijer E.J., Howell M.D. (2009). Standardised metrics for global surgical surveillance. Lancet.

[87] Cox J.A., Vlieghe E., Mendelson M., Wertheim H., Ndegwa L., Villegas M.V., Gould I., Levy Hara G. (2017). Antibiotic stewardship in low- and middle-income countries: The same but different?. Clin. Microbiol. Infect. Off. Publ. Eur. Soc. Clin. Microbiol. Infect. Dis..

[88] Sartelli M., Labricciosa F.M., Barbadoro P., Pagani L., Ansaloni L., Brink A.J., Carlet J., Khanna A., Chichom-Mefire A., Coccolini F. (2017). The Global Alliance for Infections in Surgery: Defining a model for antimicrobial stewardship-results from an international cross-sectional survey. World J. Emerg. Surg..

[89] Rivers E., Nguyen B., Havstad S., Ressler J., Muzzin A., Knoblich B., Peterson E., Tomlanovich M., Early Goal-Directed Therapy Collaborative Group (2001). Early goal-directed therapy in the treatment of severe sepsis and septic shock. N. Engl. J. Med..

[90] Rusconi A.M., Bossi I., Lampard J.G., Szava-Kovats M., Bellone A., Lang E. (2015). Early goal-directed therapy vs usual care in the treatment of severe sepsis and septic shock: A systematic review and meta-analysis. Intern. Emerg. Med..

[91] Peake S.L., Delaney A., Bailey M., Bellomo R., Cameron P.A., Cooper D.J., Higgins A.M., Holdgate A., ARISE Investigators, ANZICS Clinical Trials Group (2014). Goal-directed resuscitation for patients with early septic shock. N. Engl. J. Med..

[92] Yealy D.M., Kellum J.A., Huang D.T., Barnato A.E., Weissfeld L.A., Pike F., Terndrup T., Wang H.E., Hou P.C., ProCESS Investigators (2014). A randomized trial of protocol-based care for early septic shock. N. Engl. J. Med..

[93] Mouncey P.R., Osborn T.M., Power G.S., Harrison D.A., Sadique M.Z., Grieve R.D., Jahan R., Harvey S.E., Bell D., Bion J.F. (2015). Trial of early, goal-directed resuscitation for septic shock. N. Engl. J. Med..

[94] Sartelli M., Abu-Zidan F.M., Labricciosa F.M., Kluger Y., Coccolini F., Ansaloni L., Leppäniemi A., Kirkpatrick A.W., Tolonen M., Tranà C. (2019). Physiological parameters for Prognosis in Abdominal Sepsis (PIPAS) Study: A WSES observational study. World J. Emerg. Surg..

[95] Levy M.M., Evans L.E., Rhodes A. (2018). The Surviving Sepsis Campaign Bundle: 2018 update. Intensive Care Med..

[96] Alhazzani W., Møller M.H., Arabi Y.M., Loeb M., Gong M.N., Fan E., Oczkowski S., Levy M.M., Derde L., Dzierba A. (2020). Surviving Sepsis Campaign: Guidelines on the Management of Critically Ill Adults with Coronavirus Disease 2019 (COVID-19). Crit. Care Med..

[97] La Via L., Vasile F., Perna F., Zawadka M. (2024). Prediction of fluid responsiveness in critical care: Current evidence and future perspective. Trends Anaesth. Crit. Care.

[98] Papali A., McCurdy M.T., Calvello E.J.B. (2015). A “three delays” model for severe sepsis in resource-limited countries. J. Crit. Care.

[99] Seymour C.W., Rosengart M.R. (2015). Septic Shock: Advances in Diagnosis and Treatment. JAMA.

[100] Marik P.E., Byrne L., van Haren F. (2020). Fluid resuscitation in sepsis: The great 30 mL per kg hoax. J. Thorac. Dis..

[101] Maitland K., George E.C., Evans J.A., Kiguli S., Olupot-Olupot P., Akech S.O., Opoka R.O., Engoru C., Nyeko R., Mtove G. (2013). Exploring mechanisms of excess mortality with early fluid resuscitation: Insights from the FEAST trial. BMC Med..

[102] Keijzers G., Macdonald S.P.J., Udy A.A., Arendts G., Bailey M., Bellomo R., Blecher G.E., Burcham J., Coggins A.R., Delaney A. (2020). The Australasian Resuscitation In Sepsis Evaluation: Fluids or vasopressors in emergency department sepsis (ARISE FLUIDS), a multi-centre observational study describing current practice in Australia and New Zealand. Emerg. Med. Australas..

[103] Annane D., Renault A., Brun-Buisson C., Megarbane B., Quenot J.-P., Siami S., Cariou A., Forceville X., Schwebel C., Martin C. (2018). Hydrocortisone plus Fludrocortisone for Adults with Septic Shock. N. Engl. J. Med..

[104] Dellinger R.P., Bagshaw S.M., Antonelli M., Foster D.M., Klein D.J., Marshall J.C., Palevsky P.M., Weisberg L.S., Schorr C.A., Trzeciak S. (2018). Effect of Targeted Polymyxin B Hemoperfusion on 28-Day Mortality in Patients With Septic Shock and Elevated Endotoxin Level: The EUPHRATES Randomized Clinical Trial. JAMA.

[105] Venkatesh B., Finfer S., Cohen J., Rajbhandari D., Arabi Y., Bellomo R., Billot L., Correa M., Glass P., Harward M. (2018). Adjunctive Glucocorticoid Therapy in Patients with Septic Shock. N. Engl. J. Med..

[106] Rochwerg B., Oczkowski S.J., Siemieniuk R.A.C., Agoritsas T., Belley-Cote E., D’Aragon F., Duan E., English S., Gossack-Keenan K., Alghuroba M. (2018). Corticosteroids in Sepsis: An Updated Systematic Review and Meta-Analysis. Crit. Care Med..

[107] Lamontagne F., Rochwerg B., Lytvyn L., Guyatt G.H., Møller M.H., Annane D., Kho M.E., Adhikari N.K.J., Machado F., Vandvik P.O. (2018). Corticosteroid therapy for sepsis: A clinical practice guideline. BMJ.

[108] Alejandria M.M., Lansang M.A.D., Dans L.F., Mantaring J.B. (2013). Intravenous immunoglobulin for treating sepsis, severe sepsis and septic shock. Cochrane Database Syst. Rev..

[109] Busani S., Damiani E., Cavazzuti I., Donati A., Girardis M. (2016). Intravenous immunoglobulin in septic shock: Review of the mechanisms of action and meta-analysis of the clinical effectiveness. Minerva Anestesiol..

[110] Shankar-Hari M., Spencer J., Sewell W.A., Rowan K.M., Singer M. (2012). Bench-to-bedside review: Immunoglobulin therapy for sepsis-biological plausibility from a critical care perspective. Crit. Care.

[111] Fan E., Del Sorbo L., Goligher E.C., Hodgson C.L., Munshi L., Walkey A.J., Adhikari N.K.J., Amato M.B.P., Branson R., Brower R.G. (2017). An Official American Thoracic Society/European Society of Intensive Care Medicine/Society of Critical Care Medicine Clinical Practice Guideline: Mechanical Ventilation in Adult Patients with Acute Respiratory Distress Syndrome. Am. J. Respir. Crit. Care Med..

[112] Brower R.G., Matthay M.A., Morris A., Schoenfeld D., Thompson B.T., Wheeler A., Acute Respiratory Distress Syndrome Network (2000). Ventilation with lower tidal volumes as compared with traditional tidal volumes for acute lung injury and the acute respiratory distress syndrome. N. Engl. J. Med..

[113] Papali A., McCurdy M.T., Calvello E.J.B., Thiery G., Mawyin P.P.P., Hemsley J.G., Risko N., Colas L.N., Augustin M.E., Pierre E.J. (2017). Sepsis in Haiti: Prevalence, treatment, and outcomes in a Port-au-Prince referral hospital. J. Crit. Care.

[114] Kwizera A., Dünser M., Nakibuuka J. (2012). National intensive care unit bed capacity and ICU patient characteristics in a low income country. BMC Res. Notes.

[115] Murthy S., Adhikari N.K. (2013). Global health care of the critically ill in low-resource settings. Ann. Am. Thorac. Soc..

[116] Vukoja M., Riviello E., Gavrilovic S., Adhikari N.K.J., Kashyap R., Bhagwanjee S., Gajic O., Kilickaya O., CERTAIN Investigators (2014). A survey on critical care resources and practices in low- and middle-income countries. Glob. Heart.

[117] Neto A.S., Schultz M.J., Festic E. (2016). Ventilatory support of patients with sepsis or septic shock in resource-limited settings. Intensive Care Med..

[118] Arefian H., Heublein S., Scherag A., Brunkhorst F.M., Younis M.Z., Moerer O., Fischer D., Hartmann M. (2017). Hospital-related cost of sepsis: A systematic review. J. Infect..

[119] Paoli C.J., Reynolds M.A., Sinha M., Gitlin M., Crouser E. (2018). Epidemiology and Costs of Sepsis in the United States—An Analysis Based on Timing of Diagnosis and Severity Level. Crit. Care Med..

[120] Buchman T.G., Simpson S.Q., Sciarretta K.L., Finne K.P., Sowers N., Collier M., Chavan S., Oke I., Pennini M.E., Santhosh A. (2020). Sepsis Among Medicare Beneficiaries: 1. The Burdens of Sepsis, 2012-2018. Crit. Care Med..

[121] Álvaro-Meca A., Jiménez-Sousa M.A., Micheloud D., Sánchez-Lopez A., Heredia-Rodríguez M., Tamayo E., Resino S., Group of Biomedical Research in Critical Care Medicine (BioCritic) (2018). Epidemiological trends of sepsis in the twenty-first century (2000-2013): An analysis of incidence, mortality, and associated costs in Spain. Popul. Health Metr..

[122] Conde K.A.P., Silva E., Silva C.O., Ferreira E., Freitas F.G.R., Castro I., Rea-Neto A., Grion C.M.C., Moura A.D., Lobo S.M. (2013). Differences in sepsis treatment and outcomes between public and private hospitals in Brazil: A multicenter observational study. PLoS ONE.

[123] Baelani I., Jochberger S., Laimer T., Otieno D., Kabutu J., Wilson I., Baker T., Dünser M.W. (2011). Availability of critical care resources to treat patients with severe sepsis or septic shock in Africa: A self-reported, continent-wide survey of anaesthesia providers. Crit. Care.

[124] Cheng H.-H., Chen F.-C., Change M.-W., Kung C.-T., Cheng C.-Y., Tsai T.-C., Su C.-M., Hsiao S.-Y., Su Y.-C., Chen W.-L. (2018). Difference between elderly and non-elderly patients in using serum lactate level to predict mortality caused by sepsis in the emergency department. Medicine.

[125] Thwaites C.L., Lundeg G., Dondorp A.M., Sepsis in Resource-Limited Settings–Expert Consensus Recommendations Group of the European Society of Intensive Care Medicine (ESICM) and the Mahidol-Oxford Research Unit (MORU) in Bangkok, Thailand (2016). Infection management in patients with sepsis and septic shock in resource-limited settings. Intensive Care Med..

[126] Iwashyna T.J., Cooke C.R., Wunsch H., Kahn J.M. (2012). Population burden of long-term survivorship after severe sepsis in older Americans. J. Am. Geriatr. Soc..

[127] Winters B.D., Eberlein M., Leung J., Needham D.M., Pronovost P.J., Sevransky J.E. (2010). Long-term mortality and quality of life in sepsis: A systematic review. Crit. Care Med..

[128] Prescott H.C., Langa K.M., Iwashyna T.J. (2015). Readmission diagnoses after hospitalization for severe sepsis and other acute medical conditions. JAMA.

[129] Schuler A., Wulf D.A., Lu Y., Iwashyna T.J., Escobar G.J., Shah N.H., Liu V.X. (2018). The Impact of Acute Organ Dysfunction on Long-Term Survival in Sepsis. Crit. Care Med..

[130] Cameron J.I., Chu L.M., Matte A., Tomlinson G., Chan L., Thomas C., Friedrich J.O., Mehta S., Lamontagne F., Levasseur M. (2016). One-Year Outcomes in Caregivers of Critically Ill Patients. N. Engl. J. Med..

[131] Wintermann G.-B., Weidner K., Strauß B., Rosendahl J., Petrowski K. (2016). Predictors of posttraumatic stress and quality of life in family members of chronically critically ill patients after intensive care. Ann. Intensive Care.

[132] Allegranzi B., Bagheri Nejad S., Combescure C., Graafmans W., Attar H., Donaldson L., Pittet D. (2011). Burden of endemic health-care-associated infection in developing countries: Systematic review and meta-analysis. Lancet.

[133] Kushitor M.K., Boatemaa S. (2018). The double burden of disease and the challenge of health access: Evidence from Access, Bottlenecks, Cost and Equity facility survey in Ghana. PLoS ONE.

[134] Lagu T., Rothberg M.B., Shieh M.-S., Pekow P.S., Steingrub J.S., Lindenauer P.K. (2012). Hospitalizations, costs, and outcomes of severe sepsis in the United States 2003 to 2007. Crit. Care Med..

[135] Hall M.J., Williams S.N., DeFrances C.J., Golosinskiy A. (2011). Inpatient care for septicemia or sepsis: A challenge for patients and hospitals. NCHS Data Brief.

[136] Phua J., Koh Y., Du B., Tang Y.-Q., Divatia J.V., Tan C.C., Gomersall C.D., Faruq M.O., Shrestha B.R., Binh N.G. (2011). Management of severe sepsis in patients admitted to Asian intensive care units: Prospective cohort study. BMJ.

[137] Torio C.M., Andrews R.M. (2006). National Inpatient Hospital Costs: The Most Expensive Conditions by Payer, 2011. Healthcare Cost and Utilization Project (HCUP) Statistical Briefs.

[138] Semler M.W., Weavind L., Hooper M.H., Rice T.W., Gowda S.S., Nadas A., Song Y., Martin J.B., Bernard G.R., Wheeler A.P. (2015). An Electronic Tool for the Evaluation and Treatment of Sepsis in the ICU: A Randomized Controlled Trial. Crit. Care Med..

[139] Rudd K.E., Kissoon N., Limmathurotsakul D., Bory S., Mutahunga B., Seymour C.W., Angus D.C., West T.E., Cecconi M., Forrest P. (2018). The global burden of sepsis: Barriers and potential solutions. Crit. Care.

[140] Scheer C.S., Fuchs C., Kuhn S.-O., Vollmer M., Rehberg S., Friesecke S., Abel P., Balau V., Bandt C., Meissner K. (2017). Quality Improvement Initiative for Severe Sepsis and Septic Shock Reduces 90-Day Mortality: A 7.5-Year Observational Study. Crit. Care Med..

[141] Manaktala S., Claypool S.R. (2017). Evaluating the impact of a computerized surveillance algorithm and decision support system on sepsis mortality. J. Am. Med. Inform. Assoc..

[142] Shimabukuro D.W., Barton C.W., Feldman M.D., Mataraso S.J., Das R. (2017). Effect of a machine learning-based severe sepsis prediction algorithm on patient survival and hospital length of stay: A randomised clinical trial. BMJ Open Respir. Res..

[143] Molyneux E., Ahmad S., Robertson A. (2006). Improved triage and emergency care for children reduces inpatient mortality in a resource-constrained setting. Bull. World Health Organ..

[144] Kissoon N., Carapetis J. (2015). Pediatric sepsis in the developing world. J. Infect..

[145] Rosenthal V.D., Maki D.G., Mehta Y., Leblebicioglu H., Memish Z.A., Al-Mousa H.H., Balkhy H., Hu B., Alvarez-Moreno C., Medeiros E.A. (2014). International Nosocomial Infection Control Consortium (INICC) report, data summary of 43 countries for 2007–2012. Device-associated module. Am. J. Infect. Control.

[146] World Health Organization (2016). Guidelines on Core Components of Infection Prevention and Control Programmes at the National and Acute Health Care Facility Level. WHO Guidelines Approved by the Guidelines Review Committee.

[147] Storr J., Twyman A., Zingg W., Damani N., Kilpatrick C., Reilly J., Price L., Egger M., Grayson M.L., Kelley E. (2017). Core components for effective infection prevention and control programmes: New WHO evidence-based recommendations. Antimicrob. Resist. Infect. Control.

[148] Allegranzi B., Sax H., Bengaly L., Riebet H., Minta D.K., Chraiti M., Sokona F.M., Gayet-Ageron A., Bonnabry P., Pittet D. (2010). Successful implementation of the World Health Organization hand hygiene improvement strategy in a referral hospital in Mali, Africa. Infect. Control Hosp. Epidemiol..

[149] Luangasanatip N., Hongsuwan M., Limmathurotsakul D., Lubell Y., Lee A.S., Harbarth S., Day N.P.J., Graves N., Cooper B.S. (2015). Comparative efficacy of interventions to promote hand hygiene in hospital: Systematic review and network meta-analysis. BMJ.

[150] Pittet D., Allegranzi B., Boyce J., World Health Organization World Alliance for Patient Safety First Global Patient Safety Challenge Core Group of Experts (2009). The World Health Organization Guidelines on Hand Hygiene in Health Care and their consensus recommendations. Infect. Control HosEpidemiol..

[151] Dellit T.H., Owens R.C., McGowan J.E., Gerding D.N., Weinstein R.A., Burke J.P., Huskins W.C., Paterson D.L., Fishman N.O., Carpenter C.F. (2007). Infectious Diseases Society of America and the Society for Healthcare Epidemiology of America guidelines for developing an institutional program to enhance antimicrobial stewardship. Clin. Infect. Dis. Off. Publ. Infect. Dis. Soc. Am..

[152] Barlam T.F., Cosgrove S.E., Abbo L.M., MacDougall C., Schuetz A.N., Septimus E.J., Srinivasan A., Dellit T.H., Falck-Ytter Y.T., Fishman N.O. (2016). Implementing an Antibiotic Stewardship Program: Guidelines by the Infectious Diseases Society of America and the Society for Healthcare Epidemiology of America. Clin. Infect. Dis. Off. Publ. Infect. Dis. Soc. Am..

[153] Patel S.J., Oliveira A.P., Zhou J.J., Alba L., Furuya E.Y., Weisenberg S.A., Jia H., Clock S.A., Kubin C.J., Jenkins S.G. (2014). Risk factors and outcomes of infections caused by extremely drug-resistant gram-negative bacilli in patients hospitalized in intensive care units. Am. J. Infect. Control.

[154] Charani E., Smith I., Skodvin B., Perozziello A., Lucet J.-C., Lescure F.-X., Birgand G., Poda A., Ahmad R., Singh S. (2019). Investigating the cultural and contextual determinants of antimicrobial stewardship programmes across low-, middle- and high-income countries—A qualitative study. PLoS ONE.

[155] Gelband H., Laxminarayan R. (2015). Tackling antimicrobial resistance at global and local scales. Trends Microbiol..

[156] Ferrer R., Artigas A., Levy M.M., Blanco J., González-Díaz G., Garnacho-Montero J., Ibáñez J., Palencia E., Quintana M., de la Torre-Prados M.V. (2008). Improvement in process of care and outcome after a multicenter severe sepsis educational program in Spain. JAMA.

[157] Reinhart K., Daniels R., Kissoon N., Machado F.R., Schachter R.D., Finfer S. (2017). Recognizing Sepsis as a Global Health Priority—A WHO Resolution. N. Engl. J. Med..

[158] Tupchong K., Koyfman A., Foran M. (2015). Sepsis, severe sepsis, and septic shock: A review of the literature. Afr. J. Emerg. Med..

[159] Bonet M., Nogueira Pileggi V., Rijken M.J., Coomarasamy A., Lissauer D., Souza J.P., Gülmezoglu A.M. (2017). Towards a consensus definition of maternal sepsis: Results of a systematic review and expert consultation. Reprod. Health.

[160] Dünser M.W., Festic E., Dondorp A., Kissoon N., Ganbat T., Kwizera A., Haniffa R., Baker T., Schultz M.J., Global Intensive Care Working Group of the European Society of Intensive Care Medicine (2012). Recommendations for sepsis management in resource-limited settings. Intensive Care Med..

[161] Kwizera A., Dünser M., Nakibuuka J., Ssemogerere L., Katabira E., Nyamweya N., Mugagga E., Atwine D., Nansumba M., Tumukunde J. (2016). Clinical Characteristics and Short-Term Outcomes of HIV Patients Admitted to an African Intensive Care Unit. Crit. Care Res. Pract..

[162] Schell C.O., Gerdin Wärnberg M., Hvarfner A., Höög A., Baker U., Castegren M., Baker T. (2018). The global need for essential emergency and critical care. Crit. Care.

[163] Jacob S.T., West T.E., Banura P. (2011). Fitting a square peg into a round hole: Are the current Surviving Sepsis Campaign guidelines feasible for Africa?. Crit. Care.

[164] Lubell Y., Althaus T., Blacksell S.D., Paris D.H., Mayxay M., Pan-Ngum W., White L.J., Day N.P.J., Newton P.N. (2016). Modelling the Impact and Cost-Effectiveness of Biomarker Tests as Compared with Pathogen-Specific Diagnostics in the Management of Undifferentiated Fever in Remote Tropical Settings. PLoS ONE.

[165] Pierrakos C., Vincent J.-L. (2010). Sepsis biomarkers: A review. Crit. Care.

[166] Wacker C., Prkno A., Brunkhorst F.M., Schlattmann P. (2013). Procalcitonin as a diagnostic marker for sepsis: A systematic review and meta-analysis. Lancet Infect. Dis..

[167] Rello J., Valenzuela-Sánchez F., Ruiz-Rodriguez M., Moyano S. (2017). Sepsis: A Review of Advances in Management. Adv. Ther..

[168] Rello J., van Engelen T.S.R., Alp E., Calandra T., Cattoir V., Kern W.V., Netea M.G., Nseir S., Opal S.M., van de Veerdonk F.L. (2018). Towards precision medicine in sepsis: A position paper from the European Society of Clinical Microbiology and Infectious Diseases. Clin. Microbiol. Infect. Off. Publ. Eur. Soc. Clin. Microbiol. Infect. Dis..

[169] Srinivasan L., Page G., Kirpalani H., Murray J.C., Das A., Higgins R.D., Carlo W.A., Bell E.F., Goldberg R.N., Schibler K. (2017). Genome-wide association study of sepsis in extremely premature infants. Arch. Dis. Child. Fetal Neonatal Ed..

[170] Giamarellos-Bourboulis E.J., Opal S.M. (2016). The role of genetics and antibodies in sepsis. Ann. Transl. Med..

[171] Mira J.C., Gentile L.F., Mathias B.J., Efron P.A., Brakenridge S.C., Mohr A.M., Moore F.A., Moldawer L.L. (2017). Sepsis Pathophysiology, Chronic Critical Illness, and Persistent Inflammation-Immunosuppression and Catabolism Syndrome. Crit. Care Med..

[172] Marshall J.C. (2014). Why have clinical trials in sepsis failed?. Trends Mol. Med..

[173] Opal S.M., Dellinger R.P., Vincent J.-L., Masur H., Angus D.C. (2014). The next generation of sepsis clinical trial designs: What is next after the demise of recombinant human activated protein C?. Crit. Care Med..

[174] Deutschman C.S., Tracey K.J. (2014). Sepsis: Current dogma and new perspectives. Immunity.

[175] Marik P.E., Khangoora V., Rivera R., Hooper M.H., Catravas J. (2017). Hydrocortisone, Vitamin C, and Thiamine for the Treatment of Severe Sepsis and Septic Shock: A Retrospective Before-After Study. Chest.

[176] Sanfilippo F., Martucci G., La Via L., Cuttone G., Dimarco G., Pulizzi C., Arcadipane A., Astuto M. (2021). Hemoperfusion and blood purification strategies in patients with COVID-19: A systematic review. Artif. Organs.

[177] Rimmelé T., Kellum J.A. (2011). Clinical review: Blood purification for sepsis. Crit. Care.

[178] Honore P.M., Jacobs R., Joannes-Boyau O., De Regt J., De Waele E., van Gorp V., Boer W., Verfaillie L., Spapen H.D. (2013). Newly designed CRRT membranes for sepsis and SIRS—A pragmatic approach for bedside intensivists summarizing the more recent advances: A systematic structured review. ASAIO J. Am. Soc. Artif. Intern. Organs.

[179] Hawchar F., László I., Öveges N., Trásy D., Ondrik Z., Molnar Z. (2019). Extracorporeal cytokine adsorption in septic shock: A proof of concept randomized, controlled pilot study. J. Crit. Care.

[180] Pickkers P., van der Poll T. (2019). What’s new in immunostimulating strategies in the ICU. Intensive Care Med..

[181] Talisa V.B., Yende S., Seymour C.W., Angus D.C. (2018). Arguing for Adaptive Clinical Trials in Sepsis. Front. Immunol..

[182] Coopersmith C.M., De Backer D., Deutschman C.S., Ferrer R., Lat I., Machado F.R., Martin G.S., Martin-Loeches I., Nunnally M.E., Antonelli M. (2018). Surviving sepsis campaign: Research priorities for sepsis and septic shock. Intensive Care Med..

[183] Lat I., Coopersmith C.M., De Backer D., Machado F.R., Martin G.S., Martin-Loeches I., Nunnally M.E., Prescott H.C., Schorr C., Nunnally M.E. (2021). The Surviving Sepsis Campaign: Fluid Resuscitation and Vasopressor Therapy Research Priorities in Adult Patients. Crit. Care Med..

[184] Angus D.C., van der Poll T. (2013). Severe sepsis and septic shock. N. Engl. J. Med..

[185] Laxminarayan R., Duse A., Wattal C., Zaidi A.K.M., Wertheim H.F.L., Sumpradit N., Vlieghe E., Hara G.L., Gould I.M., Goossens H. (2013). Antibiotic resistance-the need for global solutions. Lancet Infect. Dis..

[186] Kruger P., Bailey M., Bellomo R., Cooper D.J., Harward M., Higgins A., Howe B., Jones D., Joyce C., Kostner K. (2013). A multicenter randomized trial of atorvastatin therapy in intensive care patients with severe sepsis. Am. J. Respir. Crit. Care Med..

[187] Leligdowicz A., Matthay M.A. (2019). Heterogeneity in sepsis: New biological evidence with clinical applications. Crit. Care.

